# Development of a latency model for HIV-1 subtype C and the impact of long terminal repeat element genetic variation on latency reversal

**DOI:** 10.1016/j.jve.2024.100575

**Published:** 2024-12-13

**Authors:** Shreyal Maikoo, Robert-Jan Palstra, Krista L. Dong, Tokameh Mahmoudi, Thumbi Ndung'u, Paradise Madlala

**Affiliations:** a HIV Pathogenesis Programme, The Doris Duke Medical Research Institute, Nelson R. Mandela School of Medicine, University of KwaZulu-Natal, Durban, South Africa; b School of Laboratory Medicine and Medical Sciences, University of KwaZulu-Natal, Durban, South Africa; c Department of Biochemistry, Erasmus University Medical Center, PO Box 2040, 3000CA, Rotterdam, the Netherlands; d Department of Pathology, Erasmus University Medical Center, the Netherlands; e Department of Urology, Erasmus University Medical Center, the Netherlands; f Ragon Institute of Mass General, MIT and Harvard, Cambridge, MA, USA; g Massachusetts General Hospital, Infectious Disease Division, Boston, MA, USA; h Harvard Medical School, Cambridge, MA, USA; i Africa Health Research Institute, Durban, KwaZulu-Natal, South Africa; j Division of Infection and Immunity, University College London, London, United Kingdom

**Keywords:** Genetic variation, HIV-1C latency, HIV-1 subtype C, Reactivation, Transmitted founder (T/F)

## Abstract

Sub-Saharan Africa accounts for almost 70 % of people living with HIV (PLWH) worldwide, with the greatest numbers centred in South Africa where 98 % of infections are caused by subtype C (HIV-1C). However, HIV-1 subtype B (HIV-1B), prevalent in Europe and North America, has been the focus of most cure research and testing despite making up only 12 % of HIV-1 infections globally. Development of latency models for non-subtype B viruses is a necessary step to address this disproportionate focus. Furthermore, the impact of genetic variation between viral subtypes, specifically within the long terminal repeat (LTR) element of the viral transcriptional promoter on latency reversal, remains unclear. To address this scientific gap, we constructed a minimal genome retroviral vector expressing HIV-1C consensus transactivator of transcription protein (Tat) and green fluorescent protein (GFP) under the control of either HIV-1C consensus LTR (C731CC) or the transmitted/founder (T/F) LTRs derived from PLWH (C_T/F_731CC), produced corresponding LTR pseudotyped viruses using a vesicular stomatitis virus (VSV-G) pseudotyped Envelope vector and the pCMVΔR8.91 packaging vector containing HIV-1 accessory and *rev* genes. Viruses produced in this way were used to infect Jurkat E6 and primary CD4^+^ T cells *in vitro*. By enriching for latently infected cells, and treating them with different latency reversing agents, we developed an HIV-1C latency model that demonstrated that the HIV-1C consensus LTR has lower reactivation potential compared to its HIV-1B counterpart. Furthermore, HIV-1C T/F LTR pseudotyped proviral genetic variants exhibited a heterogenous reactivation response which was modulated by host cell (genetic) variation. Our data suggests that genetic variation both within and between HIV-1 subtypes influences latency reversal. Future studies should investigate the specific role of variation in host cellular environment on reactivation differences.

## Graphical abstract

**Figure:**
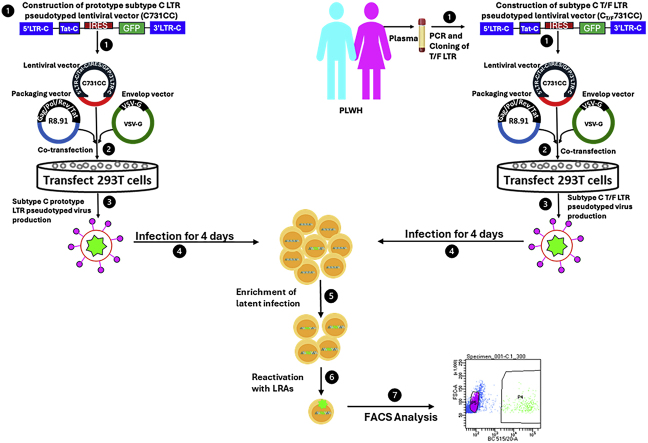

## 1 Introduction

The persistence of the latent viral reservoir despite active antiretroviral therapy (ART) is the major barrier to HIV-1 infection cure.^1^ The latent reservoir comprises of cells infected with replication competent but transcriptionally silent proviruses.^2^^,^^3^ One strategy being pursued to clear latently infected cells is to stimulate virus production from latent provirus using latency reversing agents (LRAs) (reviewed in Ref. 4).

Different classes of LRAs include histone deacetylase inhibitors (HDACis),^5^^,^^6^ protein kinase C (PKC) agonists,^7^^,^^8^ and BRG–Brahma associated factor (BAF) complex inhibitors (BAFi).^9^ HDACis hyperacetylate histones resulting in less condensed chromatin thus allowing for host transcription factors to bind the integrated proviral promoter, 5’ long terminal repeat (LTR), and induce viral gene expression.^10^ The PKC agonists such as phorbol 12-myristate 13-acetate (PMA), ionomycin (IONO) and tumor necrosis factor alpha (TNF-α) reactivate viral gene transcription through nuclear factor kappa B (NF-ĸB) signaling.^11^ However, results of clinical trials so far have not been promising. LRAs have been shown to induce differential HIV-1 reactivation, but no significant reduction in the viral reservoir size has been observed in people living with HIV-1 (PLWH) who are on ART.^5^^,^^6^^,^^10^^,^^12^^,^^13^

The HIV-1 5′ LTR is the viral promoter that drives viral gene transcription.^14^ The HIV-1 5′ LTR is identical to the 3’ LTR, and is divided into three distinct regions, U3, R and U5. Specifically, the U3 region is further divided into three functional domains that regulate HIV-1 positive sense transcription: core promoter region, enhancer region and modulatory region (reviewed in Ref. ^15^). HIV-1 subtype AE has only one NF-κB motif within the enhancer region, but most subtypes including the prototype subtype B have two, while subtype C has three to four NF-κB motifs that may translate to functional differences.^16^^,^^17^ HIV-1 subtype C (HIV-1C) strains exhibiting LTR variants with four NF-κB motifs circulate at low frequency in Brazil, Mozambique^18^ and South Africa.^19^^,^^20^ The HIV-1C viral strains exhibiting 4 NF-κB motifs were reported to be taking over the epidemic in India and were associated with higher transcription activity and viral load compared to the standard subtype C LTR variants exhibiting three NF-κB motifs.^21^ Using isolates from the FRESH acute HIV infection cohort in Durban, South Africa, we recently confirmed that HIV-1C LTR variants exhibiting four NF-κB motifs are infrequent in South Africa and that genetic variation of HIV-1C transmitted/founder (T/F) LTR impacts transcription activation and clinical disease outcomes in ART-naïve PLWH.^22^

However, the role of inter- and intra-subtype LTR genetic variation on the propensity of latency reversal has not been fully investigated. Therefore, in this study we undertook to develop a subtype C-based *in vitro* latency model, referred to as the C J-Lat cell line, by adapting methods used to develop the J-Lat model for subtype B^23^ and characterised the effect of HIV-1 LTR genetic variation on latency reactivation potential. We report the successful construction of an HIV-1C lentiviral minimal genome reporter vector expressing a green fluorescent protein (GFP) reporter and HIV-1C consensus transactivator of transcription (Tat) under the control of an HIV-1C consensus LTR (subtype C LTR-Tat-IRES-EGFP), referred to as C731CC and the development of a subtype C *in vitro* latency model (C J-Lat). Specifically, we show that this HIV-1C prototype is less sensitive to reactivation by LRAs compared to its HIV-1B counterpart, with HIV-1C T/F LTR variants exhibiting distinct different reactivation patterns. Variants with four NF-κB motifs showed lower tendency for latency reversal in Jurkat E6 and primary CD4^+^ T-cells compared to those with canonical three NF-κB motifs.

## 2 Results

### 2.1 Successful development of C J-Lat model

Genetic variation within an AP-1 of HIV-1B^24^ and a higher number of NF-κB binding sites within HIV-1C LTR were reported to be associated with rapid latency establishment and a stable viral latent state.^25^ A different study reported no gross differences among subtype specific LTR clones, both in the initial latency level and the activation response, except for subtype AE.^26^ A recent study reported that HIV-1 latency potential is influenced by intra-subtype genetic differences in the viral LTR.^27^ However, the effect of inter- and intra-subtype LTR genetic variation derived from PLWH on the propensity of latency reactivation has not been fully investigated. Therefore, we postulated that the development of a subtype C LTR-based latency model to further study subtype-specific and overall LTR-mediated genetic variation on latency reversal may be useful for future studies.

**Fig. 1:**
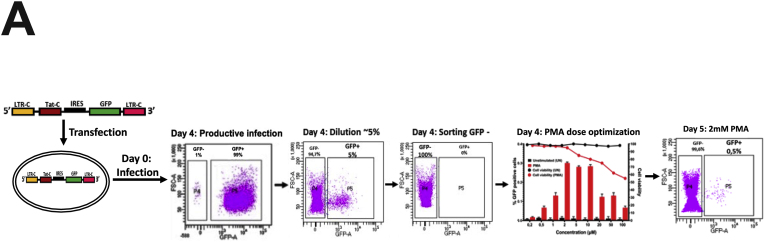

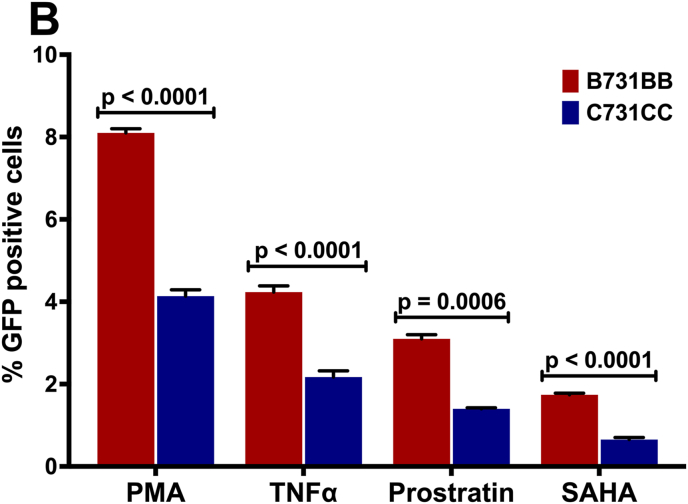

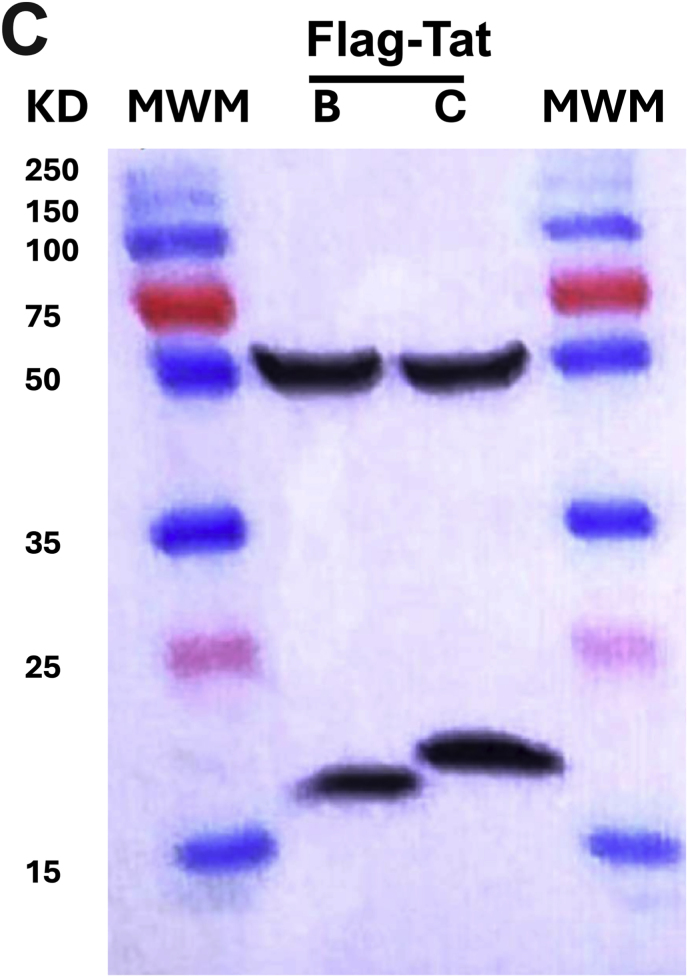

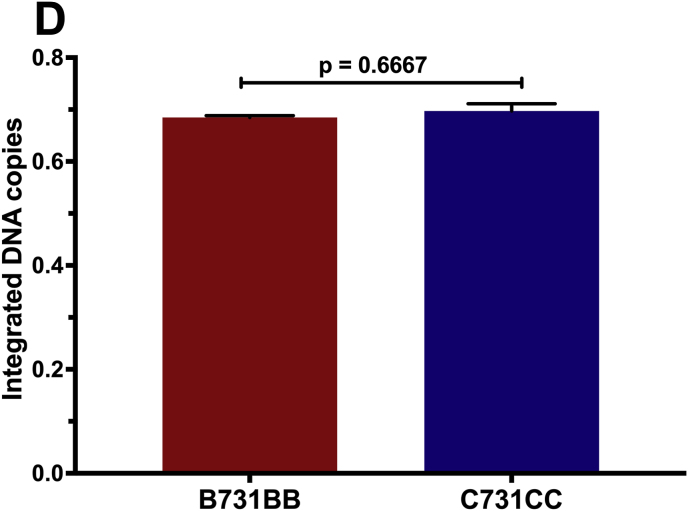
Outline of latency establishment experiment and development of C J-Lat model. A: Overview of transfection of the C731CC lentiviral vector into the Jurkat cells which resulted in productive infection (undiluted virus), as well as the dilution that gave ∼5 % GFP positive cells. We then sorted for the remaining 95 % GFP negative population (which gave 0 % GFP positive cells) and tested a range of PMA concentrations to determine the optimal dose with least toxicity to the cells. 2 μM PMA was then selected as the optimal concentration, which was then used to reactivate all latent viruses. **B:** B731BB (subtype B-red bar) was twice as more reactivatable as compared to C731CC (subtype C-blue bar) when stimulated with LRAs. **C:** Western blot demonstrating that HIV-1 Tat was equally expressed in both subtypes (Tat C is HIV-1 subtype C Tat, whilst Tat B is HIV-1 subtype B Tat). Tubulin was used as a loading control. **D:** Alu-gag PCR demonstrating that there was no significant difference in the amount of integrated HIV-1 DNA copies between B731BB and C731CC J-Lat lines.

First, we successfully developed a C J-Lat model. We show that C731CC is infectious resulting in up to 99 % of GFP positive cells and it could be serially diluted to a point of approximately 5 % GFP positive cells, which was used to enrich for latently infected cells (GFP negative cells) on the fourth day following infection and overnight reactivation with different LRAs (Fig. 1A). The concentrations of LRAs used in this study were not toxic to the cells, with 2 μM PMA yielding cell viability of 95 % (representative). Reactivation with PMA, TNF-α, prostratin and SAHA resulted in significantly lower percentages of Jurkat E6 cells latently infected with C731CC proviruses expressing GFP, with values of 0.38 %; 0.26 %; 0.11 %; and 0.04 %, compared to the percentages for GFP expressing latently infected Jurkat E6 cells with B731BB provirus, which were 0.68 %; 0.5 %; 0.2 %; and 0.11 % respectively (Fig. 1B). Western blot analysis demonstrated that the expression levels of B731BB Tat (Tat B) were similar to C731CC provirus Tat (Tat C) levels in Jurkat E6 (Fig. 1C). Equally, there were no differences in the amount of integrated HIV-1 DNA copies between B731BB and C731CC (Fig. 1D). The C731CC provirus has three NF-kB motifs while B731BB proviruses exhibits only two. In addition, the B731BB provirus contains a four-nucleotide AP-1 sequence (TGAC), whereas the C731CC proviruses has a seven-nucleotide AP-1 sequence (TGACACA) (Fig. S1). Taken together, our data show that at least for these prototype constructs, the HIV-1C latent provirus is approximately two-fold less responsive to reactivation with LRAs as compared to HIV-1B, suggesting that there may be important inter-subtype genetic variation responsible for these differences, although genetic differences that are not subtype specific cannot be ruled out.

### 2.2 Phylogenetic analysis and reactivation potential of PLWH-derived HIV-1C T/F LTR pseudotyped viral variants

**Fig. 2:**
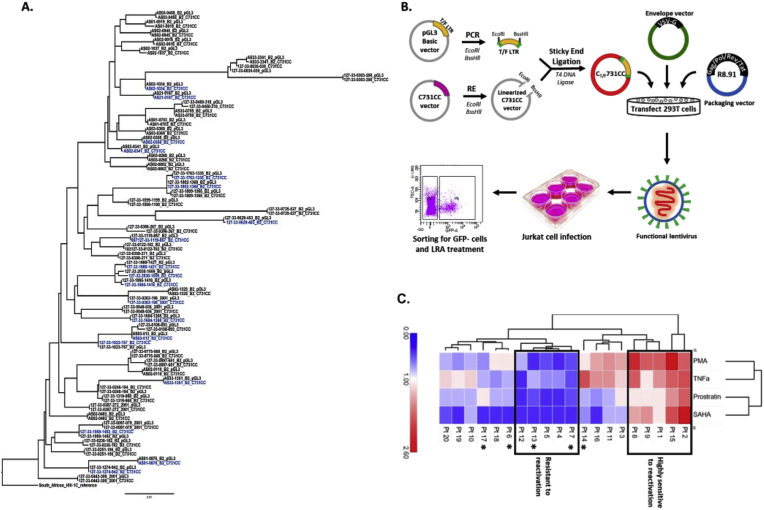
Phylogenetic analysis of participant derived HIV-1C T/F LTR sequences before and after cloning confirms successful cloning. A: Phylogenetic tree that was constructed using the online tool Phyml (http://www.hivlanl.gov) and rerooted on the South African subtype C reference sequence using Figtree software v1.4.3. The HIV-1C T/F LTR sequences obtained before cloning are denoted as pGL3, whilst those obtained after cloning are denoted as C731CC. The 20 LTR sequences highlighted in blue were randomly selected to produce the participant -derived HIV-1C T/F LTR pseudotyped viruses. The HIV-1C LTR for each participant identity (PID) from both pGL3 and C731CC clusters together thus demonstrating that the HIV-1C LTR sequence was the same before and after cloning, suggesting that cloning was successful with no sequence recombination. **B:** Overview of the methodology undertaken to produce the participant -derived HIV-1C T/F LTR pseudotyped viruses. **C:** Heatmap showing reactivation of participant LTR-Tat-GFP in latently infected Jurkat cells. Data showing differential reactivation among all 20 participant -derived HIV-1C T/F LTR pseudotyped viruses (denoted as Pt 1–20) when stimulated with LRAs. The viruses that were highly sensitive and resistant to reactivation are depicted within blocks, with the moderately reactivatable viruses remaining outside the blocks. The reactivation percentage of each T/F LTR pseudotyped virus was calculated as the fold change compared to the mean reactivation percentage of all T/F LTR pseudotyped viruses for all treatments. The heatmaps show the dendogram of the hierarchical clustering based on average linkage and the Euclidean distance. The ∗ denotes those participant sequences that contain the 4th NF-kB binding site. The heatmap in this Fig was constructed using the online tool Morpheus (https://software.broadinstitute.org/morpheus).

We recently showed that genetic variation of the HIV-1C T/F LTR impacts transcription activation potential and clinical disease outcomes in ART-naïve PLWH.^22^ However, the effect of T/F LTR genetic variation on the propensity of latency reactivation remained unknown. We hypothesized that genetic variation of PLWH-derived HIV-1C T/F LTR pseudotyped latent proviruses modulate the propensity for latency reversal. To this effect, we replaced the consensus LTR of the C731CC lentiviral vector with PLWH-derived HIV-1C T/F LTR amplified from previously published pGL3-T/F LTR recombinant plasmids.^22^ Phylogenetic analysis confirmed that PLWH-derived T/F LTR sequences belonged to HIV-1C as they rooted to South African HIV-1C reference sequence and that they were unlinked since T/F LTR sequences from different participants did not cluster together (Fig. 2A). Moreover, the T/F LTR sequences cloned into C731CC had the same branch length and clustered together with the corresponding T/F LTR sequences contained in pGL3-T/F LTR recombinant plasmids. These data confirmed that the C731CC minimal reporter vector contained the corresponding HIV-1C T/F LTR sequence.

We randomly selected 20 T/F LTR sequences to produce pseudotyped viruses and used these pseudoviruses to infect the Jurkat E6 cells (Fig. 2B). GFP negative cells were sorted from a virus dilution that yielded approximately 5 % GFP positive cells. Our data demonstrate that the 20 participant-derived HIV-1C T/F LTR pseudotyped viruses (denoted as Pt 1–20 henceforth) exhibited differential reactivation following treatment with LRAs (Fig. S2 and Table S1). Unsupervised clustering generated five distinct clusters: two clusters (Pt 2 & 15 and Pt 1, 8, & 9) showing overall higher reactivation; one cluster (Pt 3, 11, 14 & 16) showing moderately higher or mixed reactivation; one cluster (Pt 6, 10, 17, 18, 19, 20) showing moderately lower reactivation and the last cluster (Pt 4, 5, 7, 12 &13) showing overall lower reactivation compared to the average reactivation by all LRAs (Fig. 2C). This suggests that genetic variation of PLWH-derived HIV-1C T/F LTR pseudotyped latent proviruses mediate sensitivity to LRAs, resulting in differential reactivation potential.

**Fig. 3:**
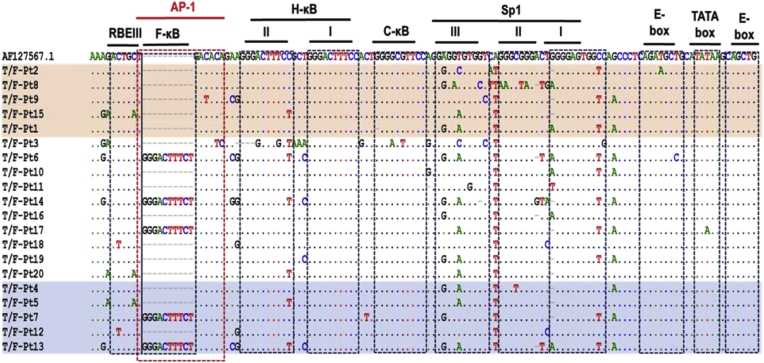
Multiple sequence alignment of participant-derived HIV-1C T/F LTR sequences. The participant-derived LTR sequences of highest (highlighted in red) and lowest (highlighted in blue) reactivating variants as determined by unsupervised clustering, were aligned against the African subtype C reference sequence, AF127567.1. The dots within the sequences represent nucleotide bases that are identical to the reference sequence while the grey dashes indicate deletions. The grey dashes within the reference sequence indicate insertions. The dotted blocks highlight the transcription factor binding sites (TFBS) within the core-enhancer and core-promoter regions. From left to right: the AP-1 binding site (enclosed within the red dotted block); the 4th NF-kB binding site (designated F-kB); standard NF-kB sites designated I and II (H- kB, II and I); subtype C specific NF-kB site (designated C-kB); Sp1 III, II and I binding sites; 5′ E-box; Tata box; and 3′ E-box.

The data from the current study shows differential reactivation of PLWH-derived HIV-1C T/F LTR pseudotyped latent proviruses in Jurkat E6 cells. Next, we investigated if differences in latency reversal may be mediated by PLWH-derived HIV-1C T/F LTR genetic variation. For this we grouped PLWH-derived HIV-1C T/F LTR pseudotyped latent proviruses into 3 groups as either highly, moderately or lowly reactivatable following treatment of Jurkat E6 with LRAs (Fig. 3). All five (100 %) highly reactivatable PLWH-derived HIV-1C T/F LTR pseudotyped latent proviral variants exhibited only three NF-κB motifs while three of out of ten (30 %) moderately and two out of five (40 %) lowly reactivatable variants had a fourth NF-κB motif, suggesting that more NF-κB motifs may be associated with lower reactivation potential. All the variants exhibited extensive genetic variation within the Sp1 motifs, which may suggest that this might be a hotspot to explain the observed phenotypic differences.

### 2.3 HIV-1C T/F LTR genetic variation modulates the propensity for latency reversal in primary cells

**Fig. 4:**
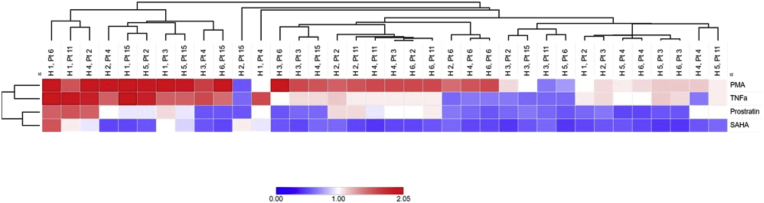
Reactivation of participant LTR-Tat-GFP in latently infected primary cells. All 6 participant (PLWH)-derived HIV-1C T/F LTR pseudotyped viruses showed differential reactivation in primary CD4^+^ T cells obtained from all six healthy donor (denoted as H 1–6) when stimulated with different LRAs. The reactivation percentage of each T/F LTR pseudotyped virus was calculated as the fold change compared to the mean reactivation percentage of all T/F LTR pseudotyped viruses for all treatments. The heatmaps show the dendogram of the hierarchical clustering based on average linkage and the Euclidean distance. The heatmaps in this Fig were constructed using the online tool Morpheus (https://software.broadinstitute.org/morpheus).

Differential reactivation of PLWH-derived HIV-1C T/F LTR pseudotyped latent proviruses in Jurkat E6 cells suggest a possible role of LTR genetic variation in latency reversal. Next, we hypothesized that genetic variation of PLWH-derived T/F LTR mediates latency reversal in primary CD4^+^ T cells.^28^ To test this hypothesis, we randomly selected six PLWH-derived HIV-1C T/F LTR pseudotyped variants−two highly (Pt 2 and Pt 15), two moderately (Pt 3 and Pt 11) and two lowly (Pt 4 and Pt 6) reactivatable in Jurkat E6 cells−from a pool of 20. We then assessed their reactivation potential in primary CD4^+^ T cells. Our data show that PLWH-derived HIV-1C T/F LTR pseudotyped latent proviruses exhibit differential reactivation potential in primary CD4^+^ T cells (Fig. 4). Specifically, our data show that the majority of HIV-1C T/F LTR pseudotyped latent proviruses were lowly to moderate reactivable in primary CD4^+^ T cells when treated with SAHA and Prostratin compared to PMA (Fig. 4 and Fig. S3). Our data suggest that while the genetic variation of HIV-1C T/F LTR pseudotyped variants contribute to heterogenous reactivation to LRAs, donor specific cellular environment may also play a role.

### 2.4 Latent HIV-1C T/F LTR pseudotyped proviruses in Jurkat E6 cells are sensitive to reactivation by LRAs targeting NF-κB and HDAC pathways albeit at differential levels

**Fig. 5:**
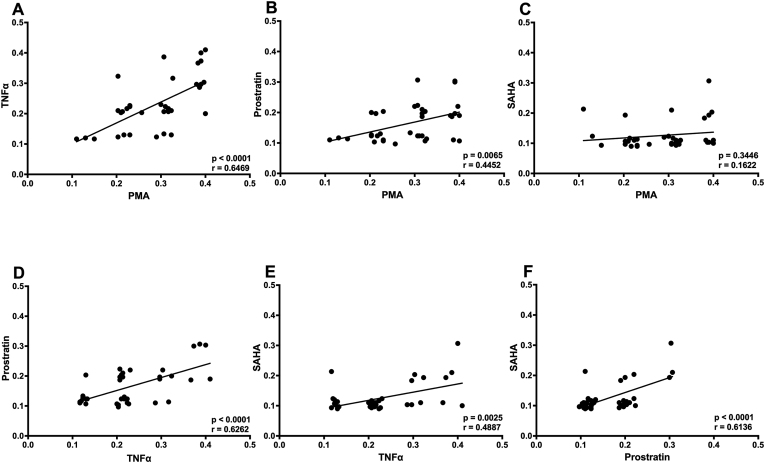
Correlation of reactivation between latency reversing agents. A: Correlation plot demonstrating significant positive correlation between PMA and TNF-α (r = 0.6469; p < 0.0001); **B:** Correlation plot demonstrating significant positive correlation between PMA and prostratin (r = 0.4452; p = 0.0065); **C:** Correlation plot demonstrating no correlation between PMA and SAHA; **D:** Correlation plot demonstrating significant positive correlation between TNF-α and prostratin (r = 0.6262; p < 0.0001); **E:** Correlation plot demonstrating significant positive correlation between TNF-α and SAHA (r = 0.4887; p = 0.0025); **F:** Correlation plot demonstrating significant positive correlation between Prostratin and SAHA (r = 0.6136; p < 0.0001).

Lastly, we hypothesized that the reactivation potentials may differ depending on the LRA used for stimulation, since each LRA works via a different pathway or mechanism to activate the latent virus (reviewed in Ref. 4). To this effect, we performed correlation analysis on the reactivation potentials observed in Jurkat cells. We observed significant positive correlation between reactivation by PMA and TNF-α (r = 0.6469; p < 0.0001) (Fig. 5A); PMA and prostratin (r = 0.4452; p = 0.0065) (Fig. 5B); TNF-α and prostratin (r = 0.6262; p < 0.0001) (Fig. 5D); TNF-α and SAHA (r = 0.4887; p = 0.0025) (Fig. 5E); as well as Prostratin and SAHA (r = 0.6136; p < 0.0001) (Fig. 5F). However, there was no correlation between reactivation by PMA and SAHA (Fig. 5C) suggesting different mechanisms of viral activation by these two LRAs.

## 3 Discussion

Sub-Saharan Africa is home to the majority of PLWH globally, with South Africa being the epicentre of the epidemic where 98 % of infections are subtype C.^29^^,^^30^ Despite its prevalence, cure research has focused on HIV-1 subtype B, which is the dominate clade in the U.S. and Europe (reviewed in Ref. ^31^). HIV-1 LTR is crucial for viral gene transcription and replication.^32^ Inter- and intra-subtype LTR genetic variation exists which translates to functional differences.^17^^,^^21^ Subtype AE has only one NF-κB binding site, while the prototype subtype B, including other subtypes, contains two and subtype C has at least three NF-κB binding sites.^14^^,^^17^ In this study, we report the development of a latency model based on HIV-1 subtype C (C J-Lat) that creates an opportunity to study impact of subtype-specific LTR genetic variation on latency reactivation potential.

Firstly, we constructed a lentiviral vector containing GFP and HIV-1C consensus Tat open reading frames both under the control of the HIV-1C promoter consensus LTR (C731CC). We then infected a culture of the Jurkat E6 cell line with viral particles generated using C731CC, R8.91 packing (containing accessory, *rev* HIV-1 subtype B genes) and VSV-G plasmids providing additional viral proteins required for efficient viral infection. A published FACS-based protocol was used to highly enrich for latently infected cells.^23^ We found that latent HIV-1C provirus was significantly less sensitive to reactivation compared to HIV-1B when C J-Lat and its subtype B counterpart J-Lat A2^23^ were treated with different LRAs. Integrated proviral DNA copies and Tat expression levels were similar between the subtypes, suggesting that the differences in reactivation potential are due to LTR genetic differences.

A previous study reported that NF-κB p50–HDAC1 complexes constitutively bind the latent HIV-1 LTR and induce histone deacetylation that results in repressive changes in the chromatin structure of the HIV-1 LTR and halts the recruitment of RNA polymerase II and transcriptional initiation.^33^ Our data showing that subtype B had a higher reactivation compared to subtype C, can be explained by subtype B containing fewer NF-κB motifs compared to subtype C and hence had fewer p50–HDAC1 repressor complexes bound at its LTR. Furthermore, less reactivation for subtype C in the current study is consistent with a previous report stating that an increase in the NF-κB motif copy number stabilizes the latent state.^34^ In addition, it has been shown that the AP-1 binding site which is upstream of the NF-κB motifs within the HIV-1 LTR, promotes HIV-1 latent infection although its deletion significantly decreases latency establishment.^24^ Additionally, the enhanced reactivation potential of subtype B can also be attributed to its four-nucleotide AP-1 sequence while the subtype C contains a seven-nucleotide AP-1 sequence which confers a stable latent viral state. However, we can't exclude that differences in HIV-1 integration sites into the human genome between the subtypes contributes to the observed differential subtype LTR transcription activities.^35^ Additionally, other outcomes may have been obtained by utilising a packing plasmid based on subtype C, with its own accessory and *rev* genes, rather than the R8.91 plasmid containing subtype B genes.

Concurrent with our previous report that genetic variation of HIV-1C T/F LTR impacts transcription activation and clinical disease outcome,^22^ in the current study we show that the PLWH-derived HIV-1C T/F LTR pseudotyped proviral variants exhibit differential reactivation potential. Specifically, our findings show three distinct groups of highly, moderately, and lowly reactivatable proviruses. Interestingly, all highly reactivatable latent HIV-1C T/F LTR pseudotyped proviruses exhibited only three NF-κB motifs while some of the moderately and lowly reactivatable proviruses exhibited the four NF-κB binding sites. However, the presence of the fourth NF-κB (F-κB) site was not exclusively associated with less reactivation potential, especially in CD4^+^ T cells, as some of the moderately and lowly reactivable HIV-1C T/F LTR pseudotyped proviruses exhibited three NF-κB motifs. All HIV-1C T/F LTRs tested exhibited variation within Sp1 binding sites. A previous study reported that specific mutations that were introduced in the Sp1 sites abolished any ability of latent viruses to be reactivated, indicating that the transcription factor Sp1 is essential for reactivation.^36^

PLWH-derived HIV-1C T/F LTR pseudotyped latent proviruses that were either highly or moderate or lowly reactivatable in Jurkat E6 cells, did not always behave the same in primary CD4^+^ cells from six healthy donors. Overall, there was no correlation between the reactivation potential in Jurkat E6 cells and primary CD4^+^ cells. Our data are consistent with a previous study reporting that there was no single *in vitro* cell model alone that was able to capture accurately the *ex vivo* response characteristics of latently infected T cells from patients^37^ and most cell models sensitivity to HIV-1 reactivation was skewed toward or against specific drug classes.^37^ Specifically, their data showed that PKC agonists and PHA reactivated latent HIV-1 uniformly across models, although drugs in most other classes did not.

Proviral transcriptional activity was previously shown to be associated with activating epigenetic chromatin features in linear proximity of integration site and in their inter- and intrachromosomal contact regions.^38^ The HDACi, SAHA, targets the epigenetic environment of the provirus by inhibiting deacetylation of histone tails and transcription repression.^10^ Consistent with previous reports showing that PKC agonists directly activate viral transcription through NF-ĸB signalling (reviewed in Ref. ^39^), our data shows that PKC agonists PMA, TNF-α and Prostratin used in this study have similar or related mechanisms of viral activation.

The limitations of this study include that we did not quantify the integrated proviral DNA levels of individual PLWH-derived HIV-1C T/F LTR pseudotyped proviruses although we do not expect differences based on our observations for the HIV-1B and HIV-1C prototype viruses. Furthermore, we did not analyse the integration sites, assessed the expression levels of Tat and the reactivation potential of subtype B and C consensus LTRs in primary CD4^+^ T cells. This study also used the T/F viruses, which is a single or very few viral strains that establishes infection and persist as latent reservoir.^40^ Future studies should investigate proviruses that produce infectious virus in an outgrowth experiment after long term therapy.

## 4 Conclusion

We show the successful development of a latency model based on HIV-1 subtype C and that LTR genetic variation mediates the propensity for latency reversal. Specifically, HIV-1 subtype B with only two NF-κB motifs and a four-nucleotide AP-1 sequence was significantly more sensitive to reactivation by LRAs compared to subtype C, which has three NF-κB motifs and seven-nucleotide AP-1 sequence. PLWH-derived HIV-1C T/F LTR genetic variation in Sp1 and number of NF-κB sites resulted heterogeneous latency reactivation. Therefore, future studies should systematically manipulate the NF-kB sites/AP1 sites and the Sp1 site in the same system to confirm the observations from the T/F LTR variants and determine the influence of integration sites on HIV-1C latency establishment or reversal. There is also a need to identify cellular biomarkers associated with HIV-1C latency reactivation potential. Our developed PLWH derived transmitted/founder LTR latency model provides an excellent starting point for such studies investigating the effect of subtype specific PLWH derived LTR genetic variation on latency potential.

## 5 Materials and methods

### 5.1 Ethics statement

The study was approved by the Biomedical Research Ethics Committee (BREC) of the University of KwaZulu-Natal (BREC/00002426/2021). Written informed consent was provided by all study participants.

### 5.2 Study design and participants

A total of 31 study participants from the Females Rising through Education, Support and Health (FRESH) cohort, and 19 study participants from the HIV Pathogenesis Programme (HPP) Acute Infection cohort were included in this study. FRESH is an ongoing prospective study established in 2012 in Durban, South Africa to study acute HIV-1 infection (AHI).^41^^,^^42^ FRESH recruits 18–23-year-old women without HIV who are at high risk for infection. FRESH study participants are co-enrolled in 48-week socioeconomic empowerment programme, with classes that coincide with the twice-weekly finger prick blood draw for HIV-1 RNA testing. Despite the inclusion of an intensive HIV prevention component and provision of once-daily oral pre-exposure prophylaxis (PrEP), 108 women have become HIV-1 positive since the start of the study. Between April 2013 and June 2014, the first 14 participants were diagnosed during AHI but not started on ART until they met the treatment eligibility criteria according to the South African national HIV guidelines (absolute CD4 T-cell count of ≤350 cells/mm^3^). Since July 2014, after IRB approval was obtained to provide immediate ART, 94 participants were diagnosed with AHI and started on ART within a median of 1 day following HIV-1 RNA detection.^41^ All participants who acquired HIV-1 infection underwent regular blood sampling. Plasma samples from 9 of the 14 individuals who started treatment during chronic infection, as well as from 22 individuals who started ART during AHI, were analyzed at the earliest post infection sample timepoint available (pre-ART initiation). The 5’ HIV-1C LTR was amplified and sequenced in these samples, from which 13 were randomly selected to produce the participant-derived HIV-1C T/F LTR pseudotyped viruses (12 individuals who started ART at the acute and one individual who started ART at the chronic phase) (outlined in Fig. S4).

Plasma samples were also obtained from participants in the HPP acute infection cohort in which men and women aged 18–24 years with acute HIV infection were initially enrolled. As we described previously,^22^ all individuals displayed detectable HIV-1 RNA levels during screening, had not yet seroconverted but subsequently showed an evolving Western blot pattern indicative of recent HIV-1 infection.^43^ The date of infection was estimated to be 14 days prior to screening. Blood samples were collected from participants at enrollment, 2 weeks, 4 weeks, 2-, 3- and 6-months post infection and then every 6 months thereafter. The CD4 T cell count, and viral loads were determined at each of these visits. The 5’ HIV-1C LTR was amplified and sequenced in 19 plasma samples from the HPP AI cohort obtained at first diagnosis or nearest timepoint depending on sample availability, from which 7 were randomly selected to produce the participant -derived HIV-1C T/F LTR pseudotyped viruses (outlined in Fig. 1).

### 5.3 Construction of subtype C based minimal reporter lentiviral vector “C731CC”

An HIV-1 subtype C based lentiviral vector, C731CC, was derived from pEV731^23^ by replacing the 5′ LTR, 3′ LTR, and *tat* gene of HIV-1B with subtype C counterparts. This modification ensures that both the HIV-1C Tat protein and GFP are under the control of the HIV-1C 5′ LTR (subtype C LTR-Tat-IRES-GFP). Briefly, the HIV-1C consensus 5′ LTR and 3′ LTR (amplified from the following reagent obtained through the NIH HIV Reagent Program, Division of AIDS, NIAID, NIH: Human Immunodeficiency Virus-1 (HIV-1) 93ZM74 LTR Luciferase Reporter Vector, ARP-4789, contributed by Dr. Reink Jeeninga and Dr. Ben Berkhout), are similar to the South African HIV-1C LTR consensus sequence, exhibiting 94.7 % similarity to the consensus LTR and *tat* (amplified from the following reagent obtained through the NIH HIV Reagent Program, Division of AIDS, NIAID, NIH: Plasmid pcDNA3.1(+) Expressing Isogenic Mutant Human Immunodeficiency Virus Type 1 Subtype C BL43/02 Tat (pC-Tat.BL43.CC), ARP-11785, contributed by Dr. Udaykumar Ranga) were purchased as gBlocks (Integrated DNA Technologies) and cloned into the pEV731 HIV minimal reporter genome plasmid in place of HIV-1B 5′ LTR, 3′LTR and *tat*.^35^ The name “C731CC” is derived from subtype C 5′ LTR, 731 (from pEV731), subtype C Tat and subtype C 3′ LTR. Specifically, the gBlock corresponding to the HIV-1C 5′ LTR was digested with *EcoRⅠ* and *BssHⅡ* (New England Biolabs), which were also used to digest the pEV731 vector. The gBlock corresponding to HIV-1C Tat was digested with *ClaⅠ* and *BamHⅠ* (New England Biolabs) which were also used to digest pEV731. The gBlock corresponding to HIV-1C 3′ LTR was digested with *PacⅠ* and *XhoⅠ* (New England Biolabs) and these enzymes were also used to restrict the pEV731 vector. The restriction fragments of the pEV731 plasmid were analyzed on a 1 % agarose gel and the bigger band fragment containing the linearized plasmid was extracted using the GeneJet Gel Extraction Kit (ThermoFisher Scientific) as per manufacturer's instructions. The corresponding LTRs and *tat* gBlocks were cloned into the linearized pEV731 vector lacking either 5′ LTR, Tat or 3′ LTR by ligation using 1 U of T4 DNA ligase (New England Biolabs) as per manufacturer's instructions, to generate the recombinant lentiviral minimal reporter construct for subtype C, “C731CC”. NEB® Stable Competent *E. coli* cells (High Efficiency) (New England Biolabs) were then transformed with this C731CC vector as per manufacturer's instructions and grown overnight for 14 hours at 30 °C on ampicillin agar plates. The C731CC vector (plasmid map shown in Fig. S5) was then purified from the bacterial cells using the GeneJet Plasmid Mini Prep Kit (Invitrogen) as per the manufacturer's instructions.

### 5.4 HIV-1C participant-derived transmitted/founder (T/F) LTR amplification, sequencing, and relatedness analysis

A total of 50 participant-derived HIV-1C transmitted/founder (T/F) U3R regions of the LTR contained in the recombinant pGL3 Basic vector^22^ (referred to as T/F LTR hence forth) were amplified using forward primer (LTRC_*ECORI*-F: 5′- TAA TAC GAC TCA CTA TAG GGT TGA ATT CTT TAA AAG AAA AGG GGG GAC -3′) containing the *EcoRI* site (underlined) and reverse primer (LTRC_*BssHII*-R: 5′- ATT TAG GTG ACA CTA TAG AGC TTT ATT GAG GCG CGC GCA GTG GGT T -3′) containing the *BssHII* site (underlined). The polymerase chain reaction (PCR) was performed using the Platinum™ Taq DNA Polymerase High Fidelity PCR Kit (Invitrogen), according to the manufacturer's instructions. Briefly, a PCR reaction was prepared on ice with 1X High Fidelity Buffer; 2 mM MgSO_4_; 0.2 mM dNTP Mix; 0.2 μM forward primer; 0.2 μM reverse primer; 1 ng template DNA; 1U of Platinum® Taq DNA Polymerase High Fidelity enzyme and PCR-grade water to make it up to a final reaction volume of 25 μL. PCR cycling conditions included an initial denaturation for 5 minutes at 94 °C, followed by 35 cycles of denaturation at 94 °C for 15 seconds; annealing at 55 °C for 30 seconds; extension at 68 °C for 30 seconds, followed by a final extension of 68 °C for 7 minutes. PCR products were then analyzed on 1 % agarose gel and purified using the QIAquick PCR Purification Kit (Qiagen, Valencia, CA) according to the manufacturer's instructions.

This purified PCR product and the corresponding HIV-1C T/F LTR still contained in the recombinant pGL3 Basic vector were sequenced using the BigDye™ Terminator v3.1 Cycle Sequencing Kit (ThermoFisher Scientific). Briefly, a sequencing reaction was separately prepared for each primer containing 2 μL of 0.4 μM primer (forward or reverse primer); 2 μL sequencing buffer; 1 μL of 20 ng/μL DNA template; 0.4 μL of BigDye v3.; and 3.4 μL of PCR grade water. This reaction was then centrifuged and subjected to an initial denaturation at 96 °C for 1 minute, 25 cycles of denaturation at 96 °C for 10 seconds, annealing at 50 °C for 5 seconds, and extension at 50 °C for 4 seconds. This was followed by a sequencing reaction clean up, in which 1 μL of 125 mM ethylenediaminetetraacetic acid (EDTA) (pH 8.0); 1 μL of 3 M sodium acetate (NaOAc) (pH 5.2); and 25 μL of 100 % ethanol were added to each reaction, vortexed and centrifuged for 20 minutes at 3000 rpm. The supernatant was then removed by inverting the reaction and centrifuging again for 1 minute. This was followed by the addition of 35 μL of 70 % ethanol to each reaction, centrifugation for 5 minutes (3000 rpm), and removal of the supernatant again by inverting the reaction and centrifuging for another minute. The sequencing reactions were then dried at 50 °C for 5 minutes and stored at 4 °C with protection from exposure to light. These samples were then analyzed using the ABI 3130xl Genetic Analyzer. All the HIV-1 LTR sequences were assembled and analyzed using the Sequencher Program v5.0 (Gene Codes Corporation). Phylogenetic relatedness analysis to compare and evaluate the similarity between pGL3 Basic vector- and C731CC lentiviral vector-derived sequences was performed by Neighbor-Joining trees (with 1000 bootstrap replicates) using PhyML Maximum Likelihood software (https://www.hiv.lanl.gov/content/sequence/PHYML/interface.html). Branching topology was visualized in Figtree (http://tree.bio.ed.ac.uk/software/figtree). Multiple sequence alignment was done using MAFFT and visualized using BioEdit. The South African HIV-1C reference strain was obtained from the Los Alamos HIV sequence database (www.hiv.lanl.gov).

### 5.5 Generation of participant-derived T/F LTR -C731CC (C_T/F_731CC) minimal reporter constructs

The participant-derived HIV-1 T/F LTR was then cloned into the C731CC vector to create a C_T/F_731CC. Briefly, the purified HIV-1C T/F LTR PCR products and C731CC were digested with *EcoRI* and *BssHII* (New England Biolabs). The restriction fragments of the C731CC lentiviral vector were analyzed on the 1 % agarose gel and the bigger band fragment containing the linearized C731CC lentiviral vector was extracted using the GeneJet Gel Extraction Kit (ThermoFisher Scientific) as per manufacturer's instructions. The purified HIV-1C T/F LTR PCR products were cloned into the linearized C731CC lentiviral vector by ligation using 1 U of T4 DNA ligase (New England Biolabs) as per manufacturer's instructions, to generate C_T/F_731CC minimal reporter lentiviral vector. NEB® Stable Competent *E. coli* cells (High Efficiency) (New England Biolabs) were then transformed with C_T/F_731CC minimal genome reporter lentiviral vector as described above. The C_T/F_731CC minimal genome reporter lentiviral vectors were then purified from the bacterial cells using the GeneJet Plasmid Mini Prep Kit (Invitrogen) as described above. An overview of this methodology is shown in Fig. 2B.

### 5.6 Cell culture

Human embryonic kidney (HEK) 293T cells were cultured in Dulbecco's Modified Eagle's Medium (DMEM) (ThermoFisher Scientific) supplemented with 10 % fetal bovine serum (FBS), 100 μg/ml penicillin-streptomycin, and 1 % HEPES at 37 °C in a humidified 95 % air-5% CO_2_ atmosphere. Jurkat cells were grown in RPMI 1640 medium containing L-glutamine (ThermoFisher Scientific) supplemented with 10 % FBS, 100 μg/ml penicillin-streptomycin, and 1 % HEPES (to make R10 medium) at 37 °C in a humidified 95 % air-5% CO_2_ atmosphere.

### 5.7 Production of HIV-1C LTR pseudotyped viruses

The HEK 293T cells were co-transfected with the C731CC or C_T/F_731CC minimal reporter lentiviral vector (plasmid), vesicular stomatitis virus (VSV-G) plasmid to contribute the pseudotyped Envelope and pCMVΔR8.91 packaging vector which contains HIV-1B accessory and *rev* genes. Briefly, on day zero 2 × 10^6^ HEK 293T cells were seeded (in supplemented DMEM as described above) in a T75 flask to achieve ∼70 % confluency by the next day. On day one, the medium was changed to DMEM only (no supplements) and a DNA mix comprising of 6 μg C731CC vector, 2 μg VSV-G plasmid, and 4.5 μg pCMVΔR8.91 packaging vector containing accessory and *rev* HIV-1 gens in a total of 500 μL was made. A transfection mix was made with 125 μL Polyethylenimine (PEI) Transfection Reagent (ThermoFisher Scientific) and DMEM to a total of 500 μL followed by incubation at room temperature for 5 minutes. The transfection mix was then added to the DNA mix dropwise and incubated at room temperature for 20 minutes. This PEI/DNA mix was then added dropwise to the cells in the T75 flask and incubated for 6 hours at 37 °C in a humidified 95 % air-5% CO_2_ atmosphere followed by a media change to R10 medium. The pseudotyped viruses were then harvested from the supernatant of cell cultures at 72 hours post media change, filter sterilized with a 0.45 μm filter, aliquoted, and stored at −80 °C. An overview of this methodology is shown in Fig. 2B.

### 5.8 Jurkat cell infection

The Jurkat cells were grown to have at least 95 % viability and were infected with either C731CC or C_T/F_731CC pseudotyped viruses, such that approximately 5 % of cells were infected. Briefly, 4 × 10^5^ cells/mL were seeded in a 6 well plate and infected with either C731CC or C_T/F_731CC pseudotyped virus for 96 hours of incubation at 37 °C in a humidified 95 % air-5% CO_2_ atmosphere to give about 5 % GFP positive cells.

### 5.9 Flow cytometry

GFP expression was analyzed by flow cytometry. The live population was defined by forward versus side scatter profiles. Gating for SSC-H vs SSC-W and FSC-H vs FSC-W was used to exclude doublets. Cells were further gated by using forward scatter versus GFP intensity to differentiate between GFP-positive and -negative cells (gating strategy shown in Fig. S6).

The 95 % of GFP negative cell population, which represented either true negative or latently infected cells were sorted using a BD FACSAria™ Fusion Flow Cytometer (Becton Dickinson) and treated with latency reversing agents (LRAs).

### 5.10 Latency reversal

Immediately following cell sorting, each individual GFP positive cell was sorted into a corresponding well of a 96-well plate, cultured until the cells turned GFP negative indicating that viral gene expression has become latent. The cells containing the latent provirus were expanded to develop single clones containing dormant HIV-1C provirus, which exhibited no GFP expression under basal condition and 99 % when stimulated with PMA, this cell line is referred to as C J-Lat henceforth.

On the other hand, GFP negative cells were centrifuged at 1500 rpm for 10 minutes and resuspended in R10 medium. The following LRAs, 2 mM phorbol 12-myristate 13-acetate (PMA); 2 μg/mL tumor necrosis factor alpha (TNF-α); 2 μg/mL prostratin; and 1500 nM suberoylanilide hydroxamic acid (SAHA) were added. The concentrations of the LRAs used are a representative concentration that was not toxic to the cell viability as viability remained high above 90 %. This was followed by 24 hours incubation at 37 °C in a humidified 95 % air-5% CO_2_ atmosphere. The percentage reactivation was then measured as the percentage GFP positive cells by flow cytometry as described above. These experiments were performed in triplicates.

### 5.11 Western blot analysis of HIV-1B and HIV-1C expression in J-Lat and C J-Lat

The J-Lat^23^ and C J-Lat (described above) cells were lysed with IP buffer (1 % NP40, 5 % glycerol, 5 mM MgCl_2_, 1 mM EDTA, 150 mM KCl, 25 mM HEPES, pH 7.9, 0.5 mM dithiothreitol and a protease inhibitor cocktail) (Sigma Aldrich) on ice for 30 minutes. Whole-cell protein lysate was used for SDS-PAGE to detect Flag-tagged HIV-1 subtype B Tat (Tat B) and HIV-1 subtype C Tat (Tat C) with anti-Flag antibody (Sigma Aldrich) and anti-mouse IgG polyclonal HRP antibody (Bio-Rad). Tubulin was used as a loading control and PageRulerTM Plus Prestained Protein Ladder (ThermoFisher Scientific) was used to determine the sizes.

### 5.12 Measurement of the amount of integrated HIV-1 DNA copies

Quantification of the integrated HIV-1 DNA copies was performed as previously described.^44^ DNA was isolated from both J-Lat and C J-Lat cells by lysing 2 × 10^6^ cells using the AllPrep DNA/RNA Mini Kit (Qiagen) as per the manufacturer's instructions. Alu-gag PCR was then performed as follows: The first round PCR reaction was prepared on ice using 1X PCR buffer (ThermoFisher), 1.5 mM MgSO_4_ (ThermoFisher), 0.5 mM dNTPs (ThermoFisher), 1 μM Alu forward primer (5′- GCC TCC CAA AGT GCT GGG ATT ACA G- 3′), 6 μM Gag reverse primer (5′- TCG CTT TCA GGT CCC TGT TCG- 3′), 2.5 U of Platinum Taq polymerase (ThermoFisher), and 150 ng of genomic DNA. Cycling conditions were as follows: initial denaturation of 95 °C for 2 minutes, followed by 14 cycles of denaturation at 95 °C for 30 seconds; annealing at 50 °C for 30 seconds and extension at 72 °C for 210 seconds. The nested PCR reaction was then prepared on ice using 1X PCR buffer (ThermoFisher), 1.5 mM MgSO_4_ (ThermoFisher), 0.5 mM dNTPs (ThermoFisher), 0.5 μM AluGag forward primer (5′- GGT GCG AGA GCG TCA GTA T- 3′) and 0.5 μM AluGag reverse primer (5′-AGC TCC CTG CTT GCC CAT A-3′), 0.15 μM AluGag probe (6FAM-AAA ATT CGG TTA AGG CCA GGG GGA AAG AA-QSY7), 1 U of Platinum Taq polymerase (ThermoFisher), and 2 μL of Alu-gag PCR product. Cycling conditions were as follows: 95 °C for 5 minutes, followed by 50 cycles of 95 °C for 10 seconds and 60 °C for 30 seconds. Human ribonuclease (RNase) P was used for normalization (ThermoFisher). The experiment were performed in three independent replicates.

### 5.13 Primary CD4^+^ T-cell isolation and infection

Peripheral blood mononuclear cells (PBMCs) were isolated from healthy donors by Ficoll gradient followed by primary CD4^+^ T-cell isolation by negative selection and magnetic separation using the human CD4^+^ T Cell Isolation kit (Miltenyi Biotec) according to the manufacturer's instructions. Isolated CD4^+^ T-cells were then rested in R10 medium at a concentration of 1 × 10^6^ cells/mL for 3 hours at 37 °C in a humidified 95 % air-5% CO_2_ atmosphere before HIV-1 infection. The CD4^+^ T-cells were then centrifuged at 800×*g* and resuspended in 200 μL of viral supernatant in a 15 mL conical centrifuge tube. The spinoculation was then carried out for 2 hours at 1200×*g* at room temperature. After spinoculation, the cells were cultured in R10 medium supplemented with 5 mM Saquinavir to prevent residual spreading infection for 72 hours at 37 °C in a humidified 95 % air-5% CO_2_ atmosphere. Saquinavir was obtained through the AIDS Research and Reference Reagent Program, Division of AIDS, NIAID, NIH. The cells were then treated with LRAs in presence of 30 mM Raltegravir (obtained from the AIDS Research and Reference Reagent Program, Division of AIDS, NIAID, NIH) for 24 hours, followed by measurement of GFP positive cells by flow cytometry as described above, to determine the percentage reactivation. These experiments were performed in triplicates. The 2 μM concentration of the PMA used is a representative concentration that was not toxic to the cells as viability remained high above 99 %.

### 5.14 Statistical and heatmap analysis

Statistical analysis was performed using GraphPad Prism 10 software. The statistical significances for the B731BB and C731CC latency reactivation comparisons were determined using an unpaired T-test, while that for integrated DNA copy measurements were determined using a non-parametric test. Linear regression analysis was used to determine the correlation coefficient and statistical significance of the correlation between the different LRAs. A p-value ≤0.05 was considered statistically significant. The heatmaps were constructed using the online tool Morpheus (https://software.broadinstitute.org/morpheus). The reactivation percentage of each T/F LTR pseudotyped virus was calculated as the fold change compared to the mean reactivation percentage of all T/F LTR pseudotyped viruses for all treatments. The heatmaps show the dendogram of the hierarchical clustering based on average linkage and the Euclidean distance.

## CRediT authorship contribution statement

**Shreyal Maikoo:** Writing – review & editing, Writing – original draft, Formal analysis, Conceptualization. **Robert-Jan Palstra:** Writing – review & editing, Methodology, Formal analysis. **Krista L. Dong:** Writing – review & editing, Resources. **Tokameh Mahmoudi:** Writing – review & editing, Resources, Methodology, Funding acquisition, Conceptualization. **Thumbi Ndung'u:** Writing – review & editing, Resources, Funding acquisition, Formal analysis, Conceptualization. **Paradise Madlala:** Writing – review & editing, Writing – original draft, Visualization, Validation, Supervision, Resources, Project administration, Methodology, Investigation, Funding acquisition, Formal analysis, Data curation, Conceptualization.

## Conflict of interest

The authors declare no conflict of interest.

## Data Availability

Data will be made available on request.

## Appendix A Supplementary data

Table S1 Raw reactivation values of patient LTR-Tat-GFP in latently infected Jurkat cells as shown in heatmap in Fig. 2C. Table S1

Multimedia component 2 Fig S1: Sequence alignment of consensus HIV-1 subtype B and C LTR core enhancer. The HIV-1 subtype B consensus 5′ LTR contains a four-nucleotide AP-1 motif just upstream of the NF-κB element, while the HIV-1 subtype C consensus 5′ LTR contains an extension of this AP-1 motif to seven nucleotides (highlighted in yellow). Fig S2: Bar graph depicting reactivation of patient LTR-Tat-GFP in latently infected Jurkat cells. All 20 patient-derived HIV-1C T/F LTR pseudotyped viruses (denoted as Pt 1–20) reactivated significantly following addition of the LRAs alone or in combination (denoted by +) compared to the unstimulated control (black bars denoted by -). Furthermore, there was differential reactivation among the patient-derived HIV-1C T/F LTR pseudotyped viruses when stimulated with different LRAs. The two highly reactivating viruses from Pt 2 and Pt 15 are denoted by the turquoise upward arrows; two moderately reactivating viruses from Pt 3 and Pt 11 are denoted by the maroon sideward arrows; and two low reactivating viruses from Pt 4 and Pt 6 are denoted by the orange downward arrows. **A**: Reactivation potentials of all 20 viruses upon PMA stimulation. **B**: Reactivation potentials of all 20 viruses upon TNF-α stimulation. **C**: Reactivation potentials of all 20 viruses upon prostratin stimulation. **D**: Reactivation potentials of all 20 viruses upon SAHA stimulation. Fig S3: Bar graph depicting reactivation of patient LTR-Tat-GFP in latently infected primary cells. All 6 patient-derived HIV-1C T/F LTR pseudotyped viruses reactivated significantly above the threshold dotted line following addition of the latency reversing agents compared to the unstimulated control (black bars) in all six healthy donor primary cells. PMA stimulation is denoted by red bars, TNF-α stimulation is denoted by blue bars, prostratin stimulation is denoted by green bars, and SAHA stimulation is denoted by purple bars. There was differential reactivation among the patient-derived HIV-1C T/F LTR pseudotyped viruses when stimulated with different LRAs in all six healthy donor primary cells. Fig S4: South African acute HIV-1 infection cohort design and the number of participants from each cohort used to generate HIV-1C T/F LTR sequences. Initiated ART at acute refers to the study participants that initiated treatment shortly after viral RNA detection. Initiated ART at chronic refers to the study participants who initiated treatment when their CD4 T cell count was below 500 cells/μL according to South African national treatment guidelines at the time. Fig S5: Plasmid map of the recombinant lentiviral minimal reporter construct for subtype C, “C731CC”. Adapted from the pEV731 HIV minimal reporter genome plasmid by cloning a HIV-1C consensus LTR and tat in place of the HIV-1B LTR (both 5′ and 3′) and tat in the pEV731 lentiviral vector, respectively. In the C731CC plasmid (subtype C LTR-Tat-IRES-EGFP), the expression of HIV-1C Tat and GFP are under the control of the HIV-1C 5′ LTR. Fig S6: Gating strategy used for flow cytometry. The live population was defined by forward versus side scatter profiles (P1). Gating for SSC-H vs SSC-W (P2) and FSC-H vs FSC-W (P3) was used to exclude doublets. Cells were further gated by using forward scatter versus GFP intensity to differentiate between GFP- negative (P4) and -positive cells (P5).
