# Supplementary material for: Development of a latency model for HIV-1 subtype C and the impact of long terminal repeat element genetic variation on latency reversal
Source: J Virus Erad. 2024 Dec 13;10(4):100575. doi: 10.1016/j.jve.2024.100575 (PMC11730875; doi:10.1016/j.jve.2024.100575)
Supplement: Table S1 — Raw reactivation values of patient LTR-Tat-GFP in latently infected Jurkat cells as shown in heatmap in Fig. 2C. [file mmc1.docx]

**Supporting information:**

| PID | Unstimulated | PMA | TNF-α | Prostratin | SAHA |
| --- | --- | --- | --- | --- | --- |
| Pt 1 | 0.00 | 0.48 | 0.40 | 0.31 | 0.29 |
| Pt 2 | 0.01 | 0.49 | 0.51 | 0.49 | 0.68 |
| Pt 3 | 0.01 | 0.39 | 0.32 | 0.30 | 0.21 |
| Pt 4 | 0.01 | 0.11 | 0.20 | 0.12 | 0.11 |
| Pt 5 | 0.01 | 0.13 | 0.19 | 0.12 | 0.11 |
| Pt 6 | 0.01 | 0.29 | 0.22 | 0.21 | 0.11 |
| Pt 7 | 0.01 | 0.12 | 0.13 | 0.13 | 0.11 |
| Pt 8 | 0.01 | 0.59 | 0.49 | 0.31 | 0.39 |
| Pt 9 | 0.01 | 0.49 | 0.30 | 0.29 | 0.40 |
| Pt 10 | 0.01 | 0.21 | 0.32 | 0.23 | 0.20 |
| Pt 11 | 0.01 | 0.42 | 0.40 | 0.20 | 0.21 |
| Pt 12 | 0.02 | 0.21 | 0.12 | 0.11 | 0.11 |
| Pt 13 | 0.01 | 0.12 | 0.20 | 0.13 | 0.12 |
| Pt 14 | 0.01 | 0.31 | 0.52 | 0.20 | 0.19 |
| Pt 15 | 0.01 | 0.71 | 0.49 | 0.41 | 0.50 |
| Pt 16 | 0.02 | 0.38 | 0.42 | 0.18 | 0.12 |
| Pt 17 | 0.01 | 0.19 | 0.21 | 0.30 | 0.11 |
| Pt 18 | 0.01 | 0.29 | 0.20 | 0.21 | 0.10 |
| Pt 19 | 0.01 | 0.19 | 0.31 | 0.20 | 0.12 |
| Pt 20 | 0.00 | 0.21 | 0.32 | 0.22 | 0.13 |

**Table S1:** Raw reactivation values of patient LTR-Tat-GFP in latently infected Jurkat cells

* PID - Participant-derived HIV-1C T/F LTR pseudotyped virus ID

* Pt - Participant-derived HIV-1C T/F LTR pseudotyped virus

**Table S1**
