# Supplementary material for: Development of a latency model for HIV-1 subtype C and the impact of long terminal repeat element genetic variation on latency reversal
Source: J Virus Erad. 2024 Dec 13;10(4):100575. doi: 10.1016/j.jve.2024.100575 (PMC11730875; doi:10.1016/j.jve.2024.100575)
Supplement: Multimedia component 2 — Fig S1: Sequence alignment of consensus HIV-1 subtype B and C LTR core enhancer. The HIV-1 subtype B consensus 5′ LTR contains a four-nucleotide AP-1 motif just upstream of the NF-κB element, while the HIV-1 subtype C consensus 5′ LTR contains an extension of this AP-1 motif to seven nucleotides (highlighted in yellow). Fig S2: Bar graph depicting reactivation of patient LTR-Tat-GFP in latently infected Jurkat cells. All 20 patient-derived HIV-1C T/F LTR pseudotyped viruses (denoted as Pt 1–20) reactivated significantly following addition of the LRAs alone or in combination (denoted by +) compared to the unstimulated control (black bars denoted by -). Furthermore, there was differential reactivation among the patient-derived HIV-1C T/F LTR pseudotyped viruses when stimulated with different LRAs. The two highly reactivating viruses from Pt 2 and Pt 15 are denoted by the turquoise upward arrows; two moderately reactivating viruses from Pt 3 and Pt 11 are denoted by the maroon sideward arrows; and two low reactivating viruses from Pt 4 and Pt 6 are denoted by the orange downward arrows. A: Reactivation potentials of all 20 viruses upon PMA stimulation. B: Reactivation potentials of all 20 viruses upon TNF-α stimulation. C: Reactivation potentials of all 20 viruses upon prostratin stimulation. D: Reactivation potentials of all 20 viruses upon SAHA stimulation. Fig S3: Bar graph depicting reactivation of patient LTR-Tat-GFP in latently infected primary cells. All 6 patient-derived HIV-1C T/F LTR pseudotyped viruses reactivated significantly above the threshold dotted line following addition of the latency reversing agents compared to the unstimulated control (black bars) in all six healthy donor primary cells. PMA stimulation is denoted by red bars, TNF-α stimulation is denoted by blue bars, prostratin stimulation is denoted by green bars, and SAHA stimulation is denoted by purple bars. There was differential reactivation among the patient-derived HIV-1C T/F LTR pseudoty [file mmc2.pdf]

Supporting Information

AP-1

Subtype B consensus: TTCAAGAACTGC**TGAC**ATCGAGCT TGCT .ACA .. AGGGACTTTCCGCTGGGGACTTTCC

Subtype C consensus: GGGACTTTCTGC**TGACACA**GAAGGGACTTTCCGCTGGGACTTTCCACCG .GGGCGTTCC

Figure S1

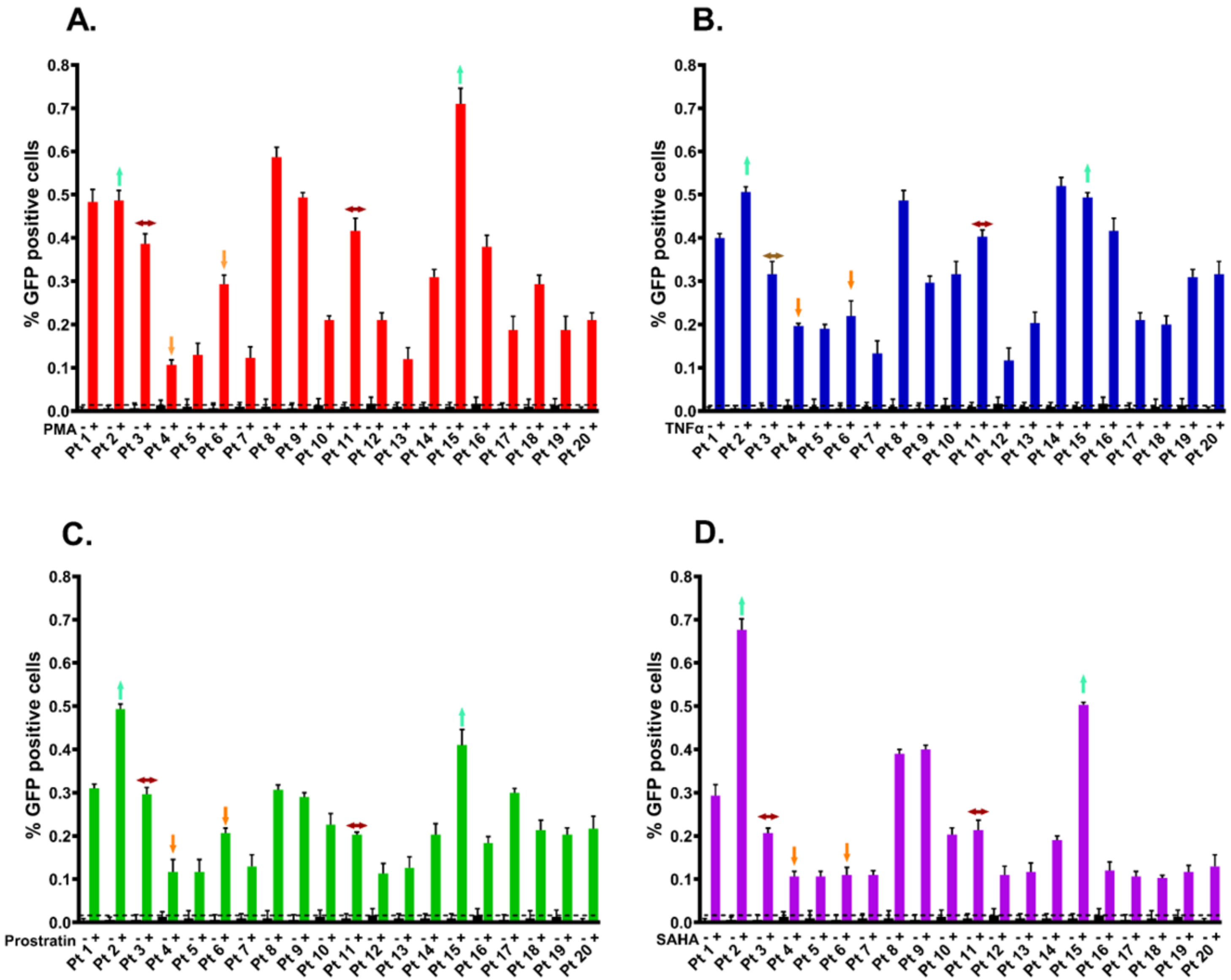
A.
B.
C.
D.
% GFP positive cells
0.0
0.1
0.2
0.3
0.4
0.5
0.6
0.7
0.8
PMA
TNFα
Prostratin
SAHA
- +
Pt 1
Pt 2
Pt 3
Pt 4
Pt 5
Pt 6
Pt 7
Pt 8
Pt 9
Pt 10
Pt 11
Pt 12
Pt 13
Pt 14
Pt 15
Pt 16
Pt 17
Pt 18
Pt 19
Pt 20

Figure S2

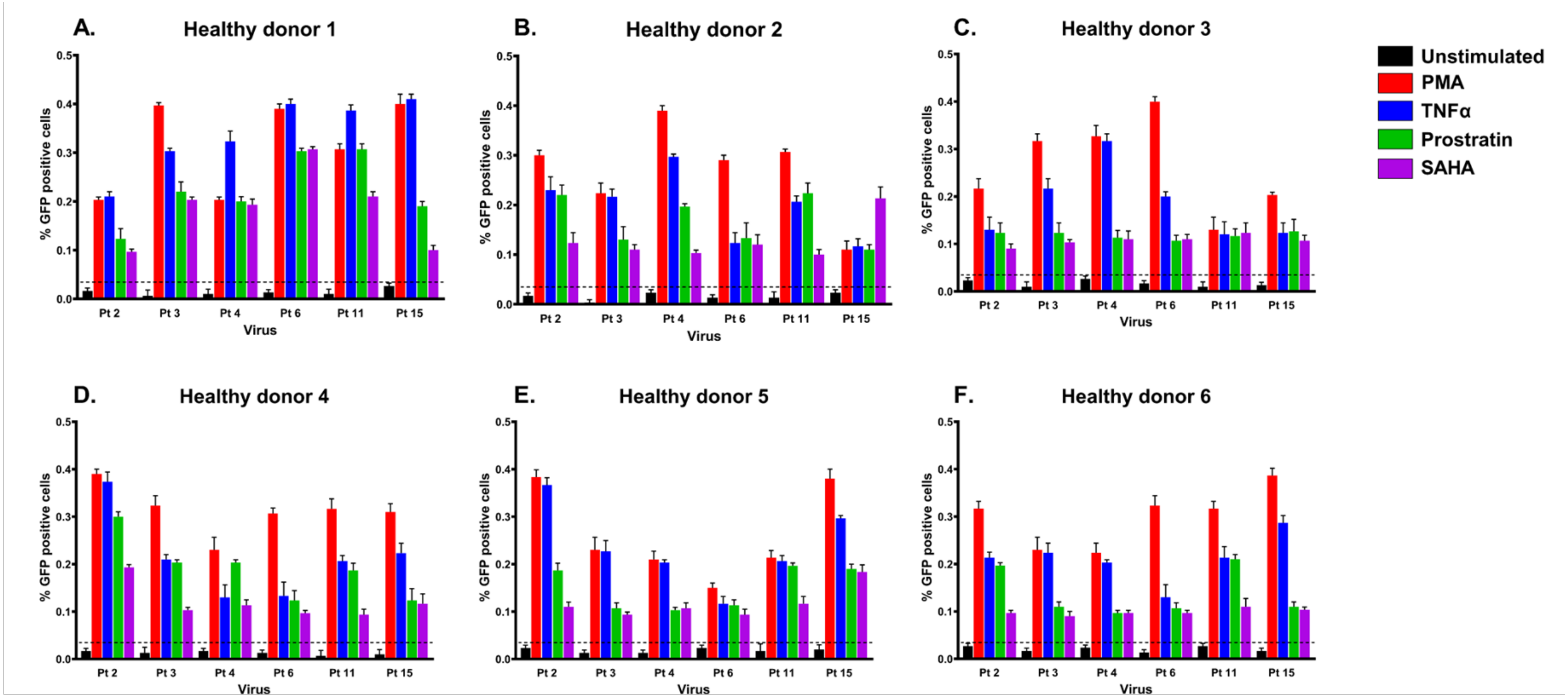
A.
Healthy donor 1
B.
Healthy donor 2
C.
Healthy donor 3
D.
Healthy donor 4
E.
Healthy donor 5
F.
Healthy donor 6
% GFP positive cells
0.0
0.1
0.2
0.3
0.4
0.5
Pt 2
Pt 3
Pt 4
Pt 6
Pt 11
Pt 15
Virus
Unstimulated
PMA
TNFα
Prostratin
SAHA

Figure S3

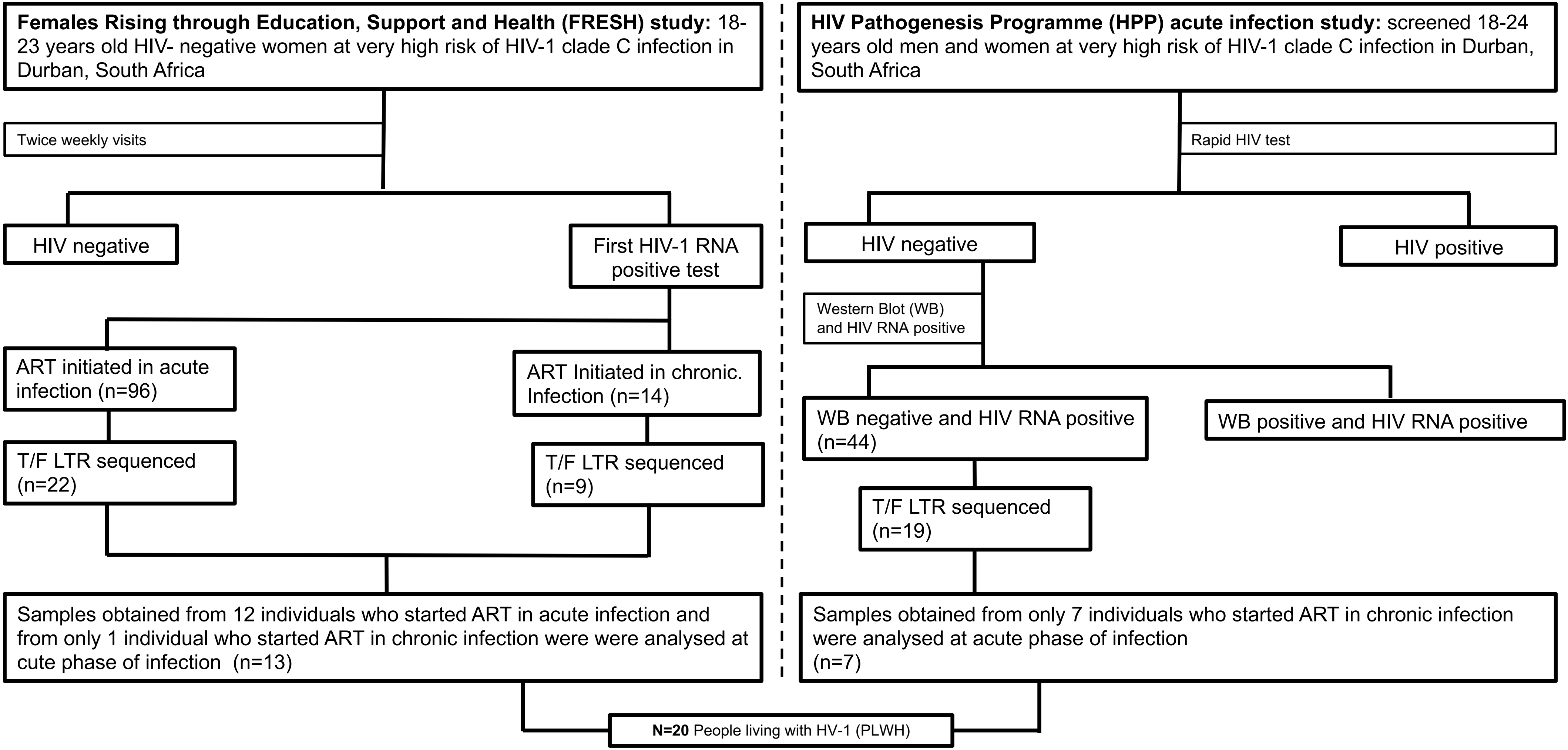
Females Rising through Education, Support and Health (FRESH) study: 18-23 years old HIV- negative women at very high risk of HIV-1 clade C infection in Durban, South Africa
Twice weekly visits
HIV negative
First HIV-1 RNA positive test
ART initiated in acute infection (n=96)
ART Initiated in chronic. Infection (n=14)
T/F LTR sequenced (n=22)
T/F LTR sequenced (n=9)
Samples obtained from 12 individuals who started ART in acute infection and from only 1 individual who started ART in chronic infection were were analysed at cute phase of infection (n=13)
HIV Pathogenesis Programme (HPP) acute infection study: screened 18-24 years old men and women at very high risk of HIV-1 clade C infection in Durban, South Africa
Rapid HIV test
HIV negative
HIV positive
Western Blot (WB) and HIV RNA positive
WB negative and HIV RNA positive (n=44)
WB positive and HIV RNA positive
T/F LTR sequenced (n=19)
Samples obtained from only 7 individuals who started ART in chronic infection were analysed at acute phase of infection (n=7)
N=20 People living with HV-1 (PLWH)

Figure S4

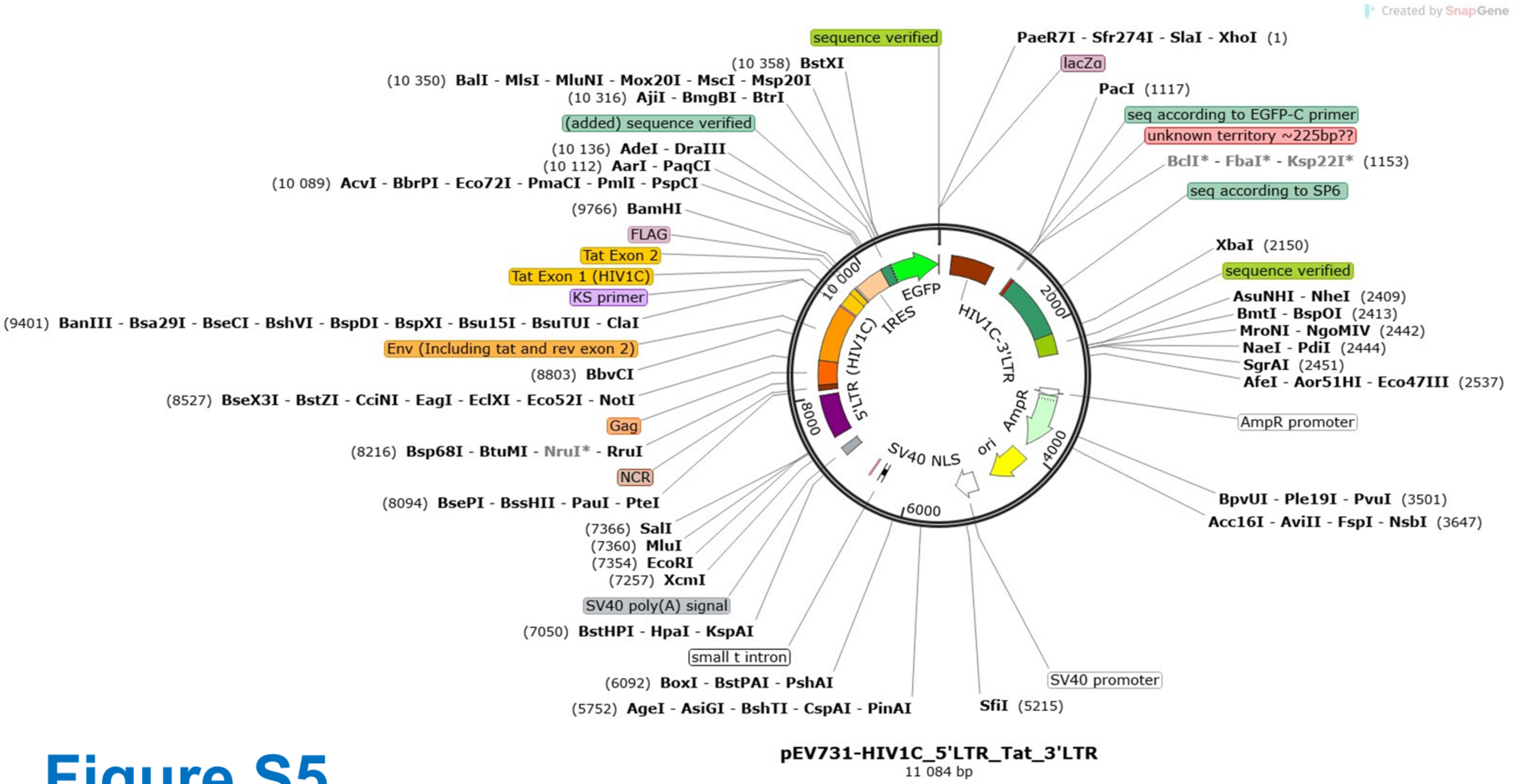

Created by SnapGene
sequence verified
PaeR7I - Sfr274I - SlaI - XhoI (1)
lacZα
PacI (1117)
seq according to EGFP-C primer
unknown territory ~225bp??
BclI* - FbaI* - Ksp22I* (1153)
seq according to SP6
XbaI (2150)
sequence verified
AsuNHI - NheI (2409)
BmtI - BspOI (2413)
MroNI - NgoMIV (2442)
NaeI - PdiI (2444)
SgrAI (2451)
AfeI - Aor51HI - Eco47III (2537)
AmpR promoter
BpvUI - Ple19I - PvuI (3501)
Acc16I - AviII - FspI - NsbI (3647)
SV40 promoter
SfiI (5215)
(5752) AgeI - AsiGI - BshTI - CspAI - PinAI
(6092) BoxI - BstPAI - PshAI
small t intron
(7050) BstHPI - HpaI - KspAI
SV40 poly(A) signal
(7257) XcmI
(7354) EcoRI
(7360) MluI
(7366) SalI
(8094) BsePI - BssHII - PauI - PteI
NCR
(8216) Bsp68I - BtuMI - NruI* - RruI
Gag
(8527) BseX3I - BstZI - CciNI - EagI - EclXI - Eco52I - NotI
(8803) BbvCI
Env (Including tat and rev exon 2)
(9401) BanIII - Bsa29I - BseCI - BshVI - BspDI - BspXI - Bsu15I - BsuTUI - ClaI
KS primer
Tat Exon 1 (HIV1C)
Tat Exon 2
FLAG
(9766) BamHI
(10 089) AcvI - BbrPI - Eco72I - PmaCI - PmlI - PspCI
(10 112) AarI - PaqCI
(10 136) AdeI - DraIII
(added) sequence verified
(10 316) AjiI - BmgBI - BtrI
(10 350) BalI - MlsI - MluNI - Mox20I - MscI - Msp20I
(10 358) BstXI
EGFP
IRES
HIV1C-3'LTR
AmpR
ori
SV40 NLS
5'LTR (HIV1C)
2000
4000
6000
8000
10 000
pEV731-HIV1C_5'LTR_Tat_3'LTR
11 084 bp

Figure S5

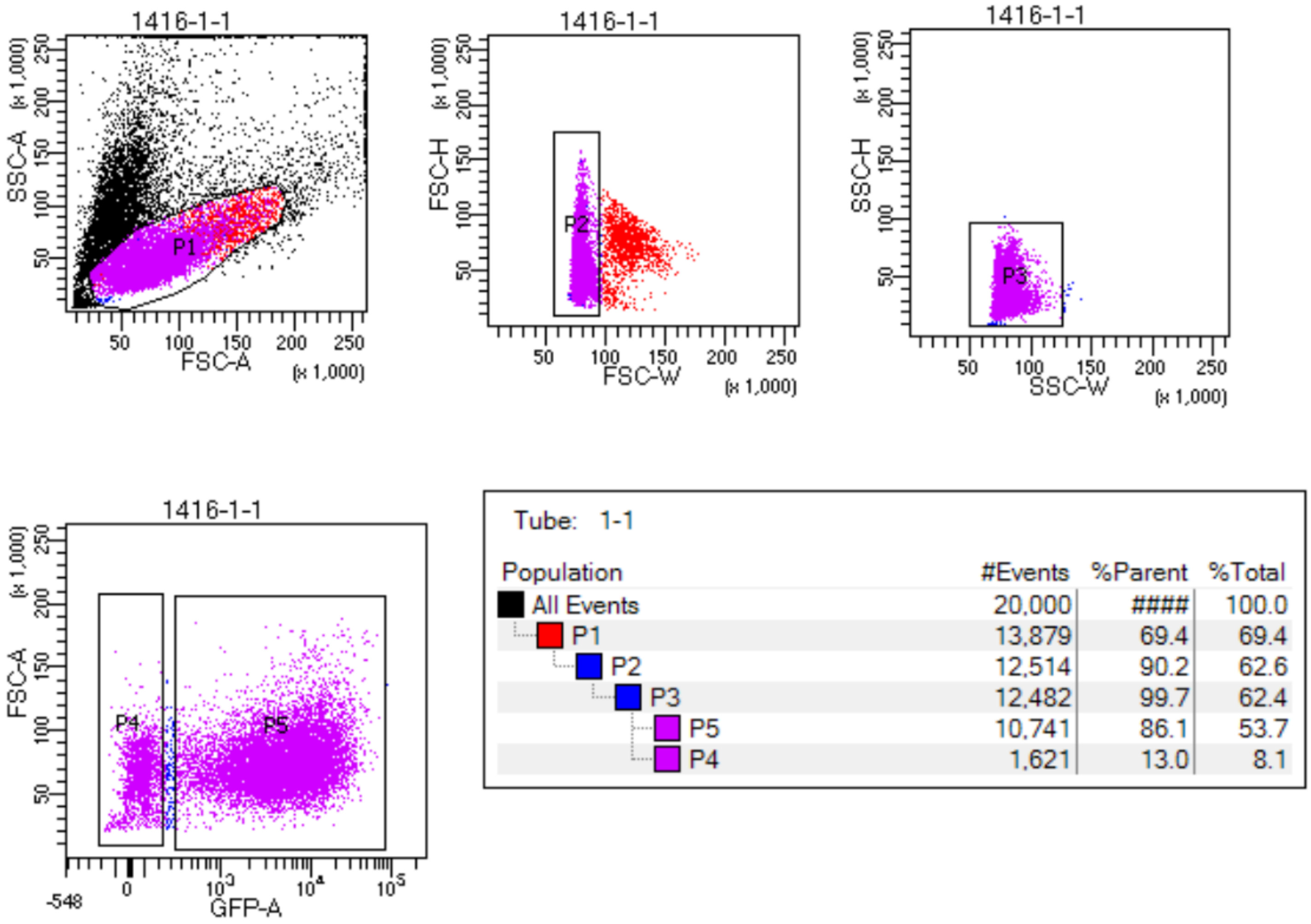

Tube: 1-1

Population	#Events	%Parent	%Total
All Events	20,000	####	100.0
P1	13,879	69.4	69.4
P2	12,514	90.2	62.6
P3	12,482	99.7	62.4
P5	10,741	86.1	53.7
P4	1,621	13.0	8.1

Figure S6
